# Evaluation of caries risk assessment practices among dental practitioners in Guangzhou, China: a cross-sectional study

**DOI:** 10.3389/froh.2024.1458188

**Published:** 2024-10-16

**Authors:** Ermin Nie, Rui Jiang, Rafiqul Islam, Xiang Li, Jiali Yu

**Affiliations:** ^1^ Department of Stomatology, First Affiliated Hospital, Sun Yat-Sen University, Guangzhou, China; ^2^ Department of Restorative Dentistry, Faculty of Dental Medicine, Hokkaido University, Sapporo, Japan

**Keywords:** dental caries, caries risk assessment, caries risk factors, caries prevention, fluoride, oral health

## Abstract

**Introduction:**

This cross-sectional study aimed to investigate dental practitioners’ knowledge and practices regarding Caries risk assessment (CRA) in routine clinical practice in Guangzhou, China.

**Methods:**

An online questionnaire was disseminated to dental practitioners to gather socio-demographic information, factors associated with CRA, the implementation of preventive treatment, and the level of awareness regarding personalized preventive treatment in relation to CRA. Statistical analyses included descriptive statistics, Chi-square tests, ANOVA, MANCOVA, linear regression, and scatter plots.

**Results and discussion:**

Out of the 695 dental practitioners who were contacted, 206 dentists participated in the online survey. However, out of the total number of dentists, 198 were successfully recruited, while the remaining 8 dentists had incomplete data in their questionnaires. 92.4% of dentists provided in-office fluoride treatments, and 73.2% held a strong belief in the correlation between current oral hygiene and tooth cavities. 23.7% of dentists evaluated caries risk on an individual basis, and a significant 41.9% never utilized a particular type of CRA. 53.5% of dentists recommended non-prescription fluoride rinses, whereas 51% advocated prescription fluoride treatments. Significant statistical relationships were found between the use of in-office fluoride and the effectiveness of restorative treatment (*P* < 0.05). Additionally, a significant association was discovered between the use of a specific form for CRA and the kind of dental school (*P* < 0.05). The study suggests that a significant number of dental practitioners in Guangzhou, China, do not utilize dedicated assessment forms for CRA in their routine professional activities. These findings highlight the im-portance of encouraging dentists to utilize CRA systems to effectively identify patients who are at risk of acquiring dental caries.

## 1 Introduction

Dental caries is a common public health problem that impacts people of every age and socioeconomic status and has a substantial effect on general well-being (1, 2). Despite scientific progress, dental caries continues to be a significant worldwide health issue, impacting a large percentage of school-aged children and nearly all adults. It is the most prevalent chronic disease among children and young individuals (3). Dental caries development is influenced by various factors such as household oral hygiene, bacterial contact, nutrition, and host reactions (4). Hence, a multifaceted preventative strategy is essential to avoid dental caries (4, 5). This method involves detecting and evaluating individuals who are likely to develop dental caries in the future using a variety of tools and diagnostic simulations (5). The conventional surgical-restorative method for treating dental caries has been substituted with a disease prevention strategy focused on risk assessment, prevention, and management (2, 6).

In order to prevent or arrest carious lesions, a systematic approach is required to assess and monitor the caries risk (CR) factors that lead to demineralization (7). Several methods have been developed to evaluate CR factors for individual patients. Treatment recommendations, such as behavioral modifications (oral hygiene and nutrition), chemical interventions (fluoride), and minimally invasive treatments, may be pursued after consideration of the patient's risk factors (7, 8). The predictive validity of a caries risk assessment (CRA) tool may be affected by the prevalence of caries and other population specific characteristics (9). Therefore, CRA tools have been developed for various populations to maximize their predictability (9).

CRA is a critical initial step in preventive treatment, necessary for the diagnosis and management of dental caries (10). CRA is also essential for determining the likelihood of developing new carious lesions or an increase in the size or activity of existing lesions during a specific period (11, 12). It is recommended that caries treatment planning includes risk assessment for each patient to establish a personalized preventive and treatment program according to current standard protocols (13–15). There are various CRA tools available, such as Cariogram (16), Caries Management by Risk Assessment (CAMBRA) (17), American Association of Pediatric Dentistry Caries Assessment Tool (AAPDCAT) (18), and American Dental Association (ADA) CRA (19). These tools consider various factors such as previous caries experience, saliva, diet, general health, fluoride exposure, and plaque to generate a prediction of the risk level for carious lesions and identify pathogenic components implicated in each patient to minimize caries occurrence through improved preventive interventions (20). Moreover, the assessment of CR factors in patients and the precise categorization of risk levels can help clinicians implement individualized caries management, resulting in appropriate recalls, increased cost-effectiveness of preventive interventions, and improved allocation of resources at both individual and societal levels (21). However, clinicians should periodically reassess the CR to ensure the effectiveness and necessity of preventive therapy, and CRA de-termination should be considered a professional service (22).

Despite the recognition of the importance of CRA among dental practitioners, studies have shown that its utilization is still low, ranging from 25% to 73% among clinicians in different countries (10, 22–24). These findings suggest that CRA implementation may depend on a variety of factors, including practitioners’ understanding of caries development, attitudes toward the condition, and proficiency in preventive measures (25). Research conducted in both developed and developing Asian countries has found varying levels of knowledge, attitudes, and practices among dental practitioners concerning CRA and management (23, 26, 27). For example, a previous study evaluated the knowledge, attitudes, and practices related to CRA among dentists in China, with participants from the central, eastern, and western regions. However, the study did not specify the specific province under investigation, leaving a knowledge gap concerning the knowledge and practices of dental practitioners in Guangzhou, China (21).

Guangzhou, located in Southern China, is a prominent city known for its rapid advancements in healthcare technology and its role as a significant center for medical education and research. The city's diverse population, combined with its modern healthcare infrastructure, makes it an ideal location for investigating the implementation of CRA in routine clinical settings. Moreover, the results of this study may also be applied to other areas in China and around the world due to Guangzhou's strategic significance as a center of healthcare innovation. Therefore, this research aims to investigate the implementation of CRA in routine clinical settings among dental practitioners in Guangzhou, China. Furthermore, the research aims to evaluate the awareness of participants about CR factors and identify the factors that are linked to their level of knowledge and utilization of CRA in their daily clinical practice. The future prospects of this study include increasing the use of CRA among dental practitioners in their daily routine practice in Guangzhou. By identifying the specific limitations, the present study can enhance dental practitioners knowledge and application of CRA in Guangzhou.

## 2 Methods

### 2.1 Ethical approval

This cross-sectional study received ethical approval (approval number 2022-424) from the First Affiliated Hospital, Sun Yat-Sen University, Guangzhou, China. The study utilized Chinese language questionnaires that were distributed to practicing dentists in Guangzhou. The questionnaires were originally developed in English and subsequently translated into Chinese. Due to the online format of the survey, participants were unable to sign a standard written informed consent form. Instead, they provided online consent to participate in the study. The survey was conducted between October 1 and November 30, 2022, with permission from the relevant authorities.

### 2.2 Sample size calculation and participant selection

The sample size for this cross-sectional study was calculated using the single population proportion formula.

$$n=\frac{Z^{2}p(1-p)}{d^{2}}$$

The following assumptions were made: the proportion (P) of individualized CRA instructions for patients was estimated to be 86% based on a previous study by Francisco et al. (26). A 95% confidence level (CI) and a marginal error (d) of 5% were also used. To account for non-response, a 5% non-response rate was added. The minimum required sample size was calculated to be 185. To account for the possibility of missing data, the sample size was increased by 10% to a final number of 203 participants.

#### 2.2.1 Participant selection

To ensure a representative sample and minimize potential biases, a stratified sampling method was employed. Licensed dental practitioners in Guangzhou were stratified based on the size of their practice (clinics/hospitals), geographic location (central/rural regions), and specialization (general dentistry/specialist).

#### 2.2.2 Recruitment process

The study population was drawn from existing contact lists of licensed dental practitioners in Guangzhou. A total of 695 dental practitioners were contacted via email. A total of 206 dentists responded to the online survey, resulting in a response rate of 29.6%. Of these, 8 questionnaires were excluded due to incomplete data, leaving 198 fully completed responses for analysis.

### 2.3 Questionnaire

The questionnaire has been developed using relevant literature (21–23, 26) and prior research to ensure it covers all the essential criteria comprehensively.

#### 2.3.1 Conceptualization

The questionnaire's content and structure were developed after a comprehensive examination of current literature and prior surveys related to CRA procedures. Key areas of focus were identified, such as sociodemographic data, caries prevention approaches, and individualized risk assessment. The questionnaire was de-signed to gather in-depth information about how dental professionals apply and interpret CRA.

#### 2.3.2 Preparation

The questionnaire comprised three main sections:

1. Sociodemographic and Professional Background:

Age, gender, type of practice, educational background, specialization, and highest degree obtained.

2. Caries Prevention Practices:

Specific preventive measures like dental sealants, fluoride applications, and patient education about oral hygiene.

3. CRA and Individualized Preventive Treatment:

Techniques and frequency of CRA practices, including the use of specialized tools and the delivery of tailored preventive care.

#### 2.3.3 Validation

Prior to its deployment, the questionnaire underwent a rigorous validation process:

• Expert Review: A group of dental research professionals examined the questionnaire to confirm its relevance and thorough coverage of the subject.
• Pilot Testing: The first version underwent pilot testing with a small group of dental practitioners to evaluate the clarity and reliability of the questions. The pilot study sought feedback on the questionnaire's length, linguistic clarity, validity, and reliability. Minor adjustments were made to the questionnaire depending on the feedback received from participants. Feedback from this phase prompted adjustments in phrasing and the answer scale to improve comprehension and precision of responses.

Response Options:

• Binary choices (Yes/No) for direct practice-related questions.
• A scaled response (Never or 0%, 1%–24%, 25%–49%, 50%–74%, 75%–99%, Every time or 100%) for questions assessing the frequency of practices.

#### 2.3.4 Data collection

The questionnaires were distributed electronically to practicing dentists in Guangzhou, facilitated by online consent due to the virtual format. The study was conducted over two months, from October 1 to November 30, 2022.

### 2.4 Statistical analysis

Descriptive statistics have been tested to precisely characterize the demographic characteristics of the participants and their replies to the questionnaire. The study investigated the correlation between socio-demographic variables and the utilization of preventive treatment for dental caries and personalized preventive treatment for CRA among dental practitioners. This was done by using Chi-square testing. An analysis of variance (ANOVA) and a multivariate analysis of covariance (MANCOVA) were used to determine significant differences and connections between different characteristics and practices related to CRA.

A multiple regression analysis was conducted to investigate the impact of variables such as gender, age, years of experience, and advanced degrees on the probability of endorsing fluoride prescriptions, chlorhexidine rinse, and sugarless or xylitol chewing gum. Scatter plots were created to visually depict the data and the connections between variables. Furthermore, we generated estimated marginal means plots to visually represent the impact of these variables on the utilization of dental explorers for lesion diagnosis and the utilization of specific forms for CRA. The statistical significance threshold was established at *p* < 0.05. The statistical analyses were conducted using SPSS software version 22.

## 3 Results

### 3.1 Sociodemographic characteristics

The study included 198 dentists, of which 39.9% were male and 60.1% were female, with a mean age of 30.42 years (SD = 7.1). A majority (61.1%) worked in public hospitals, and 93.9% graduated from public institutes. Approximately half (51.5%) were specialists, while 48.5% were general practitioners. Most dentists (58.6%) did not possess an advanced degree, with 30.8% holding a master's degree and 10.6% a PhD. The majority (78.8%) reported performing restorative treatments, and 58.6% had 3–5 years of practice experience, while 6% had more than 20 years of experience (Table 1). The demographic profile suggests a sample dominated by young, female dentists working in public hospitals. This reflects the composition of the dental workforce in Guangzhou, where public institutions play a significant role in healthcare delivery.

**Table 1:** The socio-demographic characteristics of the participants.

Characteristics	Values
Age (Mean ± SD)	30.42 ± 7.1
	Percentage (Frequency)
Gender	
Male	39.9% (79)
Female	60.1% (119)
Type of practice	
Private clinic	29.8% (59)
Public hospital	61.1% (121)
Private hospital	9.1% (18)
Type of dental school (graduation)	
Public dental school	93.9% (186)
Private dental school	6.1% (12)
Specialization	
Specialist	51.5% (102)
General practitioner	48.5% (96)
Advanced degree	
No advanced degree	58.6% (116)
Master	30.8% (6)
PhD	10.6% (21)
Performing restorative treatment	
Yes	78.8% (156)
No	21.2% (42)
Years of practice	
3–5 years	58.6% (116)
6–10 years	23.2% (46)
11–20 years	30% (15.2)
More than 20 years	6% (3.0)

### 3.2 Knowledge of caries risk factors

**Figure 1:**
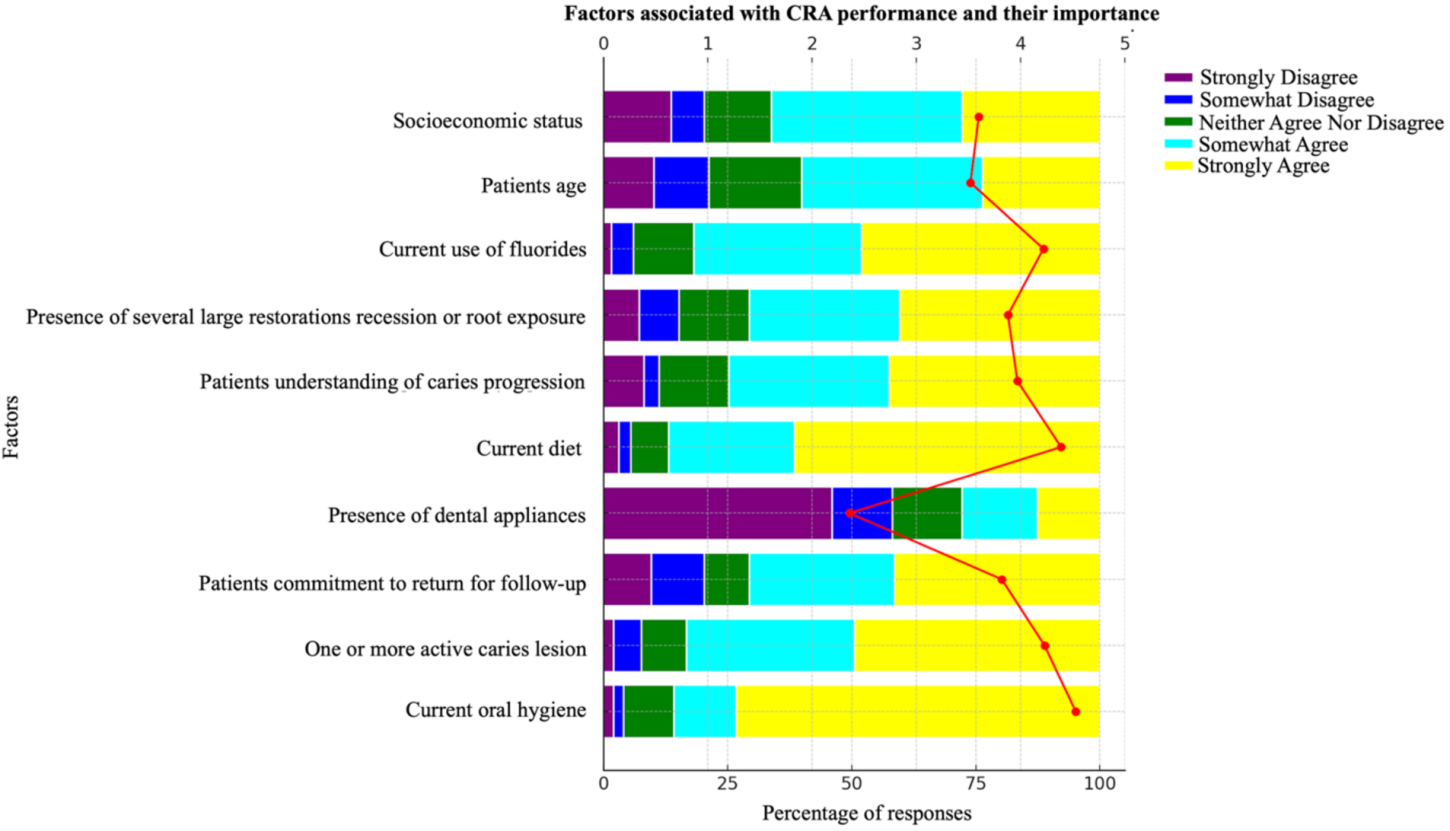
Factors related with the performance of CRA among dental practitioners in Guangzhou.

A significant majority of dentists (73.2%) strongly agreed that current oral hygiene is associated with dental caries. However, only 49.5% believed that the presence of one or more active carious lesions contributes to the development of dental caries, and just 41.4% agreed that a lack of patient commitment to follow-up appointments is a risk factor. Additionally, 46% strongly disagreed that dental appliances are associated with caries risk. The knowledge among dentists regarding other factors, such as patient age and socioeconomic status, was even less pronounced, with only 36.4% and 38.4% somewhat agreeing to their association with caries risk, respectively (Figure 1). While most dentists recognize oral hygiene as a critical factor in caries development, awareness of other risk factors, particularly patient behavior, and socioeconomic factors, is less widespread.

### 3.3 Preventive treatment practice

**Figure 2:**
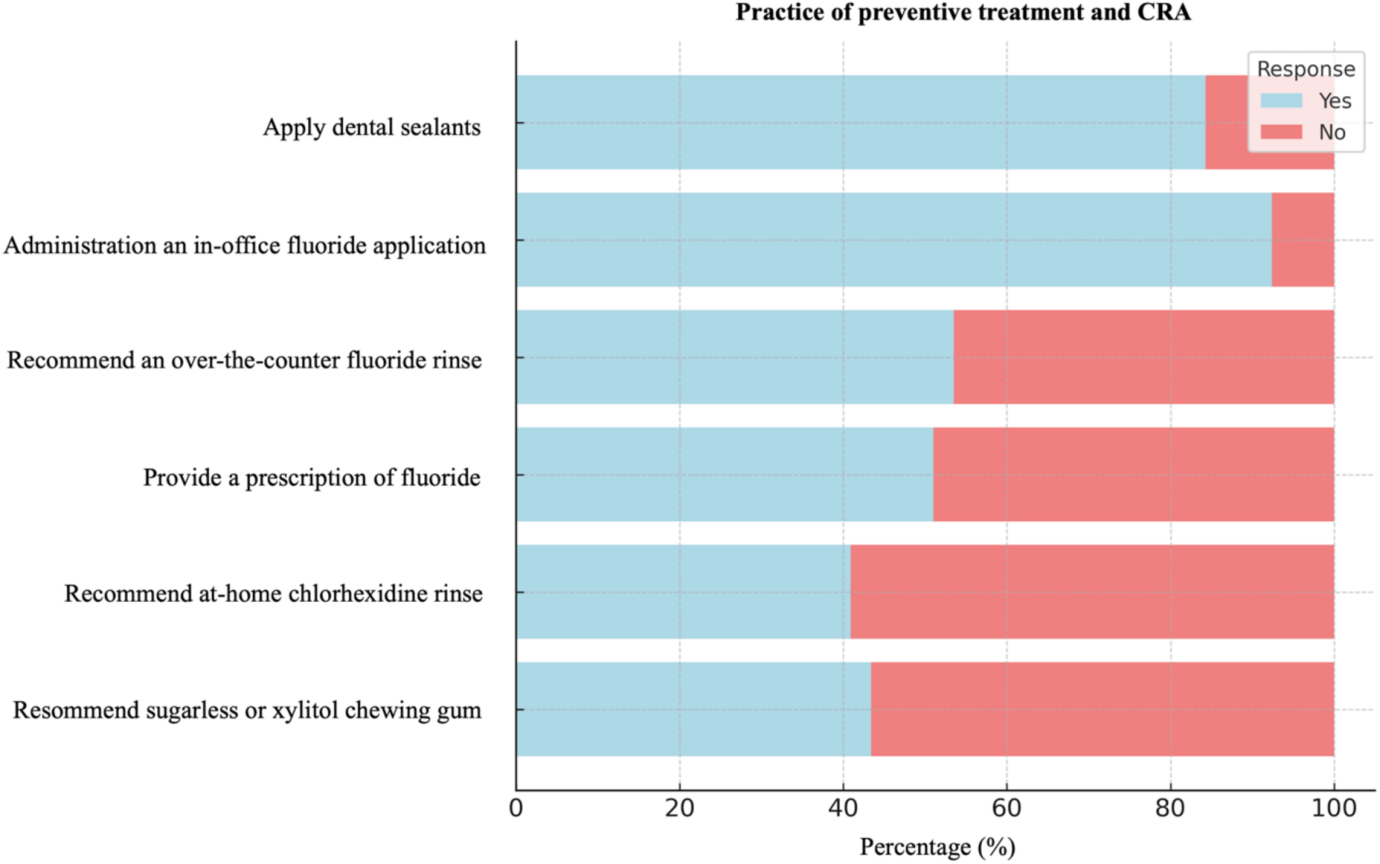
The practice of preventive treatment and CRA among dental practitioners.

The majority of dentists (84.3%) reported regularly applying dental sealants to their patients’ permanent teeth, and 92.4% administered in-office fluoride treatments. However, more than half recommended non-prescription (53.5%) and prescription fluoride (51%) to their patients. Notably, the use of at-home preventive measures, such as chlorhexidine rinse and sugarless or xylitol gum, was less common (Figure 2). Based on the results, preventive practices like sealant application and fluoride treatment are widely adopted, though recommendations for at-home care remain less frequent.

### 3.4 Individualized preventive treatment

**Figure 3:**
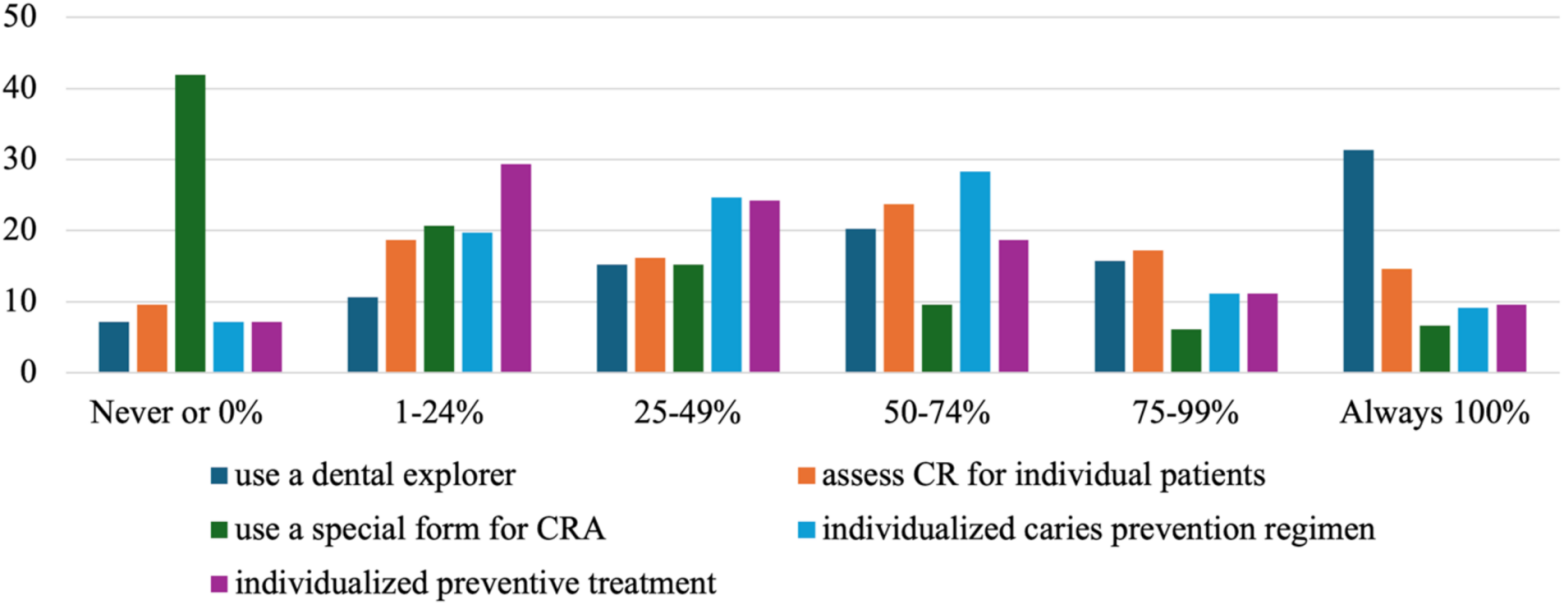
Individualized knowledge of preventive treatment related to CRA.

The use of individualized preventive treatments related to CRA was limited. Only 31.3% of dentists consistently used a dental explorer to diagnose carious lesions, and just 23.7% evaluated CR individually in 50%–74% of their patients. Notably, 41.9% never used a special form for CRA, and only 29.3% of dentists provided individualized preventive treatment to 1%–24% of their patients (Figure 3). The limited use of tools like dental explorers and special CRA forms may indicate barriers to the full implementation of CRA in clinical practice.

### 3.5 Relationship between sociodemographic characteristics and practice

**Figure 4:**
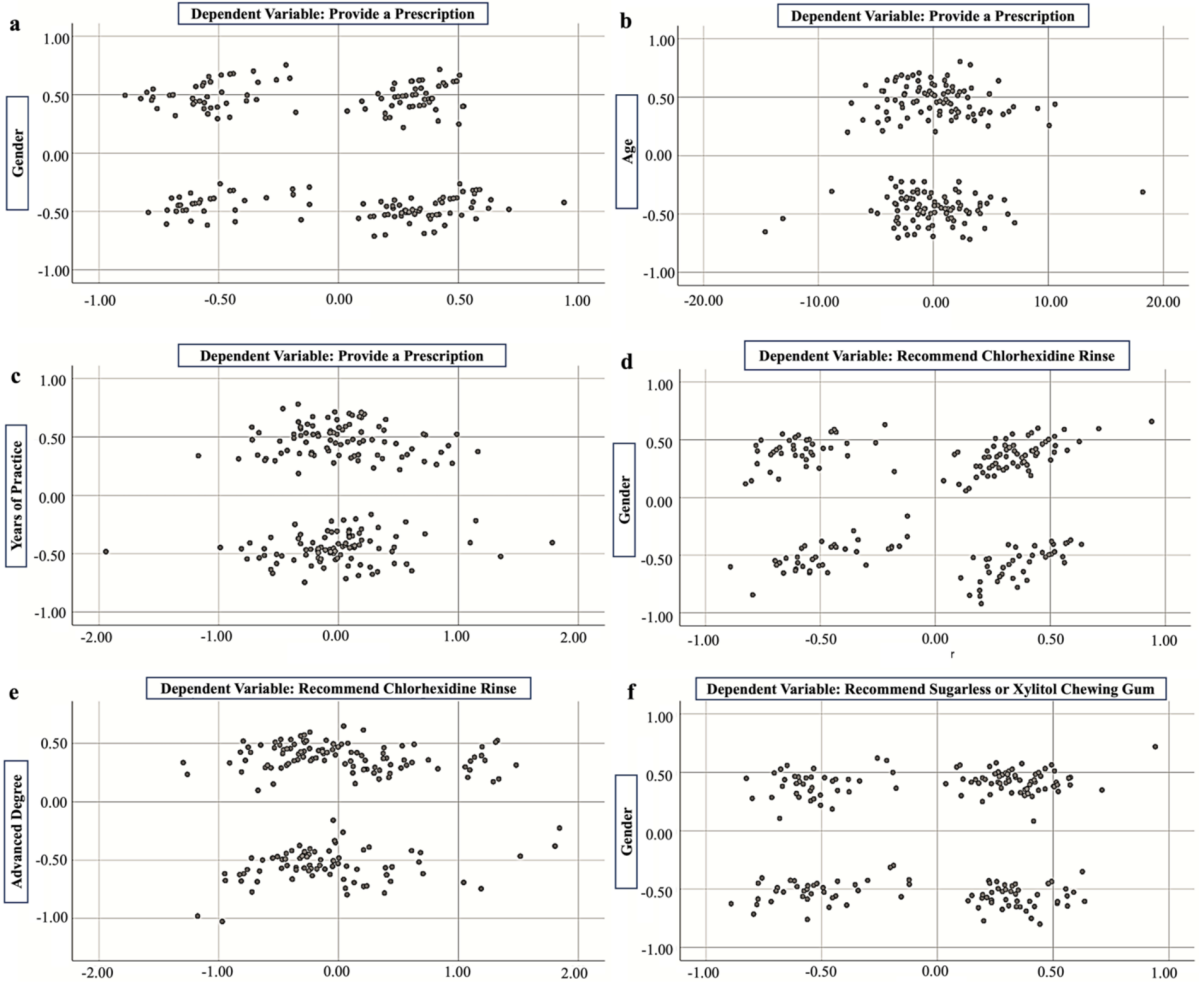
The relationship between **(a)** provide a prescription and gender, **(b)** provide a prescription and age, **(c)** provide a prescription and years of practice, **(d)** recommend chlorhexidine rinse and gender, **(e)** recommend chlorhexidine rinse and advanced degree, **(f)** recommend sugarless or xylitol chewing gum and gender.

The relationships between sociodemographic characteristics and the practice of dental caries prevention are presented in Table 2; Figure 4. Gender has had significant effects on the prescription of fluoride, recommendations for chlorhexidine rinse, and advice on sugarless or xylitol chewing gum, with male and female practitioners displaying different preferences (Figures 4a,d,f). Age and years of experience also played a critical role, with more experienced practitioners more likely to prescribe fluoride, potentially reflecting their adherence to conservative or traditional practices learned early in their careers (Figures 4b,c). Additionally, those with advanced degrees were more inclined to recommend chlorhexidine rinse, possibly due to their levels of education (Figure 4e).

**Table 2:** The relationship between sociodemographic characteristics and the practice of preventive treatment of dental caries among dental practitioners in Guangzhou, China.

	Apply dental sealants	Administration an in-office fluoride application	Recommend an over-the-counter fluoride rinse	Provide a prescription of fluoride	Recommend chlorhexidine rinse	Recommend sugarless or xylitol chewing gum
Age	2.10, (0.148)	0.29, (0.591)	0.40, (0.838)	3.37, (0.040)^a^	2.82, (0.094)	1.16, (0.283)
Gender	0.02, (0.891)	0.09, (0.772)	2.55, (0.112)	4.78, (0.030)^a^	5.79, (0.017)^a^	5.41, (0.021)^a^
Type of practice	0.73, (0.393)	3.54, (0.061)	2.85, (0.093)	3.86, (0.051)	2.35, (0.127)	0.28, (0.595)
Type of dental school	0.01, (0.921)	1.50, (0.222)	0.72, (0.398)	0.27, (0.603)	0.43, (0.511)	1.15, (0.285)
Specialization	0.16, (0.689)	0.46, (0.496)	0.10, (0.911)	0.71, (0.401)	1.52, (0.219)	2.32, (0.129)
Advanced degree	0.68, (0.411)	0.10, (0.752)	0.03, (0.858)	1.34, (0.249)	6.83, (0.010)^a^	1.46, (0.228)
Performing restorative treatment	0.08, (0.784)	6.43, (0.102)	0.03, (0.867)	0.30, (0.586)	0.01, (0.949)	0.07, (0.933)
Years of practice	0.68, (0.411)	0.04, (0.849)	1.54, (0.217)	4.27, (0.040)^a^	0.65, (0.423)	2.79, (0.096)

The level of significance (*P* < 0.05).

^a^ Indicates statistically significant difference.

### 3.6 Association between sociodemographic characteristics and individual preventive treatment

**Figure 5:**
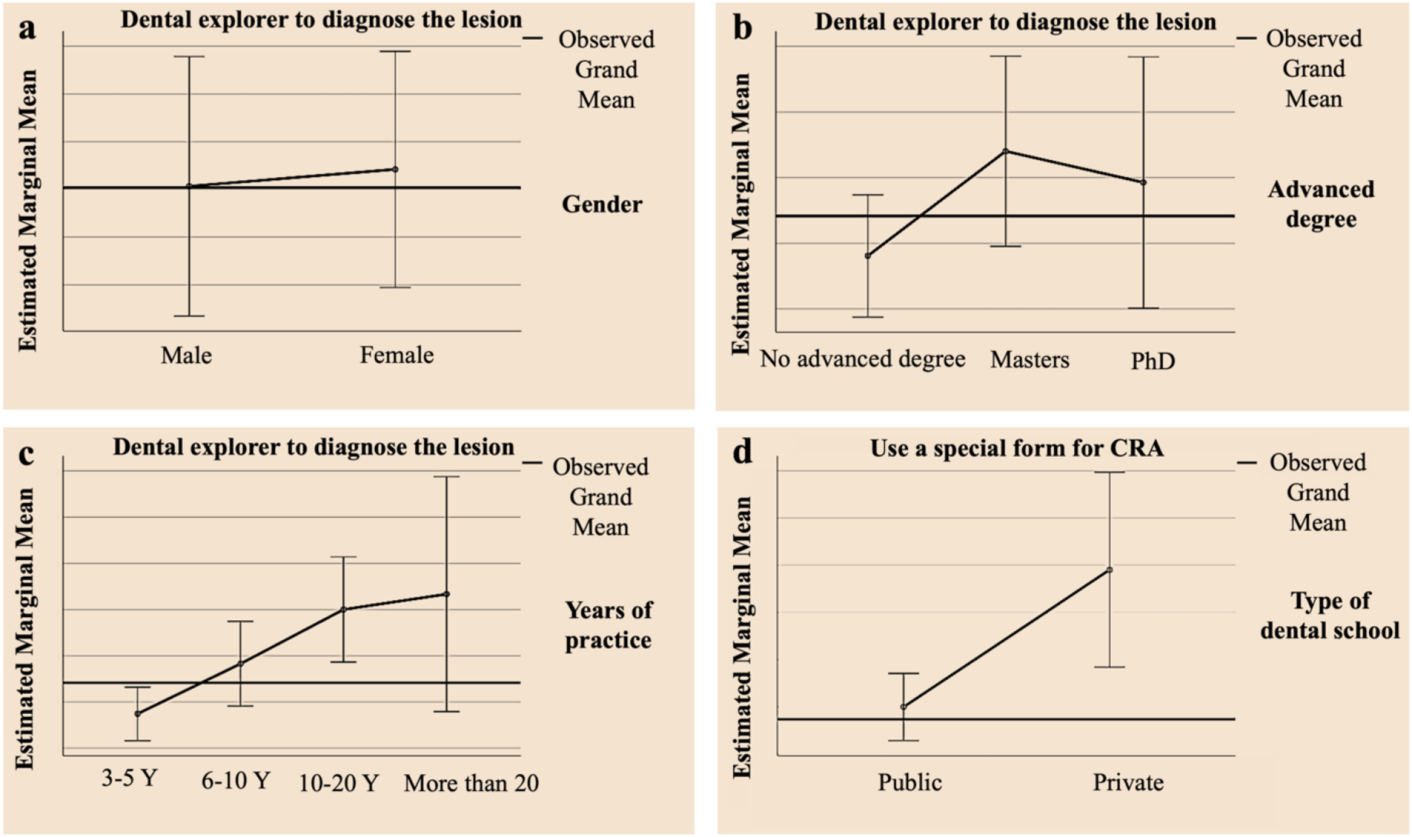
The associations between sociodemographic characteristics and individualized preventive treatment for CRA, **(a)** gender and use of dental explorer to diagnose the lesion, **(b)** advanced degree and use of dental explorer to diagnose the lesion, **(c)** years of practice and use of dental explorer to diagnose the lesion, **(d)** types of dental school and use of special form for CRA.

Table 3; Figure 5 presents the associations between sociodemographic characteristics and individualized preventive treatment for CRA. A statistically significant difference was found in the use of a dental explorer for diagnosing lesions based on gender, with male practitioners more likely to use this tool (*P* = 0.000) (Figure 5a). Additionally, the use of a specific form for CRA significantly differed depending on the type of dental school (*P* = 0.016) (Figure 5d). Practitioners with advanced degrees were significantly more likely to use a dental explorer for diagnosis (*P* = 0.008) (Figure 5b). Extensive experience also correlated significantly with the use of a dental explorer (*P* = 0.006) (Figure 5c). The results indicate that gender, type of dental school, advanced education, and extensive experience are significantly associated with the use of specific diagnostic tools and forms in CRA practices.

**Table 3:** The relationship between sociodemographic characteristics and individual preventive treatment for CRA among dental practitioners in Guangzhou.

Socio-demographic variable	Use a dental explorer to diagnose the lesion?	Do you assess CR individual patients?	Do you use a special form for CRA?	Recommend to individual caries prevention regimen?	Do you give individualized preventive treatment?
Age	0.77, (0.571)	0.54, (0.743)	0.06, (0.997)	1.16, (0.329)	1.16, (0.329)
Gender	4.82, (0.000)^a^	0.81, (0.546)	1.82, (0.110)	1.41, (0.223)	1.41, (0.223)
Type of practice	2.41, (0.380)	0.44, (0.818)	0.82, (0.536)	0.28, (0.926)	0.28, (0.926)
Type of dental school	0.79. (0.557)	0.48, (0.790)	2.87, (0.016)^a^	1.16, (0.331)	1.16, (0.331)
Specialization	1.35, (0.244)	0.78, (0.564)	0.95, (0.449)	0.52, (0.763)	0.52, (0.763)
Advanced degree	3.25, (0.008)^a^	1.09, (0.362)	1.18, (0.323)	0.51, (0.707)	0.59, (0.707)
Performing restorative treatment	0.72, (0.609)	1.02, (0.406)	1.51, (0.190)	0.49, (0.784)	0.49, (0.784)
Years of practice	3.39, (0.006)^a^	0.76, (0.576)	1.37, (0.237)	1.66, (0.147)	1.66, (0.147)

ANOVA test: the data presented are the F-test value and the level of significance (*P* < 0.05).

^a^ Indicates statistically significant difference.

## 4 Discussion

This study is the first to examine the knowledge and practices related to CRA among dental clinicians in Guangzhou, China. The study specifically examined dentists in Guangzhou, while a prior study gathered data from a more diverse group of dentists from various places in China (21). Both studies concluded that dentists in China did not commonly utilize CRA in their everyday clinical practice. In the previous survey, 35.4% of respondents used CRA in ordinary practice (21); however, the current study revealed that only 23.7% of dentists individually screened patients for CR. This discrepancy may reflect regional variations in clinical training, accessibility to CRA tools, or differences in patient demographics and demand. The present study revealed that a higher percentage of dentists provided in-office fluoride treatments, suggested over-the-counter fluoride rinses, and prescribed fluoride in some form compared to the prior study. These findings suggest an increasing recognition of fluorides role in caries prevention, likely driven by mounting evidence supporting its efficacy and possibly enhanced training in preventive dentistry. This could reveal whether there have been any changes in the knowledge and implementation of CRA among dental practitioners in China over time, and whether these changes have been uniformed across various parts of the country (28).

Interestingly, the factors prioritized by Guangzhou dentists when assessing caries risk, such as oral hygiene, diet, active caries lesions, and fluoride use, align with global trends observed in studies from the US and other countries (10, 29, 30). However, the hierarchy of risk factors varies across different studies, highlighting the influence of regional practice standards, training, and cultural perceptions of oral health (12, 31). In the present study, dentists in Guangzhou discovered the use of fluoride as another significant influence. This might be because of high priority placed on fluoride use reflects the success of public health initiatives promoting fluoride, which has been proven to reduce caries significantly (32, 33).

The possibility of dentists recommending dental sealants, in-office fluoride treatment, or over-the-counter fluoride to their patients may vary based on the percentage of patients interested in reducing the occurrence of dental caries (34). Although there is limited literature supporting the cost-effectiveness of using fluorides and sealants for caries prevention in patients, dentists may be more likely to adopt a preventive approach if patients show interest in preventing caries. Patient interest may have a stronger influence on dentists than patient clinical records (13, 34). The results of the study revealed that most dental specialists in Guangzhou recommended dental sealants (84.3%) and in-office fluoride (92.4%) applications to prevent tooth decay where dentists from Japanese and US dental practice-based research networks suggest in-office fluoride application for 21% and 37% of adult patients, respectively (10, 35). This discrepancy may be influenced by local dental practice standards, patient expectations, or the perceived cost-effectiveness of these interventions, despite the limited literature explicitly supporting their economic viability.

Notably, older and more experienced dentists were found to be more likely to prescribe fluoride, while male dentists showed a higher likelihood of recommending chlorhexidine rinses and xylitol products. These associations may reflect generational differences in training, comfort with preventive therapies, or gender-specific perceptions of treatment effectiveness (19, 20). The infrequent use of chlorhexidine rinse, despite its documented impact on *Streptococcus mutans* levels in high caries-risk patients, underscores the ongoing debate over its clinical relevance given conflicting evidence regarding its efficacy (36–38). This highlights the need for further research to clarify the role of chlorhexidine and similar preventive agents in routine dental practice.

Despite the recognized importance of CRA, only 31.3% of dentists always use a dental explorer to diagnose carious lesions, and merely 14.6% assess caries risk individually for their patients always. Additionally, a substantial 41.9% of dentists reported never using a special form for CRA. This lower percentage of non-CRA users may be due to dental clinicians lacking the time or motivation to systematically conduct a comprehensive and accurate CRA. The lack of comprehension regarding the importance of personalized preventive therapy among dentists who do not evaluate a patient's CR is also a contributing factor. Although this percentage (9.6%) appears low, it is still lower than the 31% reported by Riley et al. for practitioners from the US and Scandinavia, and considerably lower than the 74% reported by Japanese dentists who did not use CRA (10, 35). It is important to note that surveys tend to provide a more optimistic picture of the situation, as only those practitioners who are most interested in the subject tend to respond (24). Therefore, the actual percentage of practitioners who conduct CRA in daily practice was likely to be much lower, which was unfortunate since CRA was a crucial component of practicing minimal intervention in a rational manner (24). Moreover, CRA not only aids in determining the predictive risk level for new lesions in the future, but it also helps identify each pathological factor implicated in each clinical case and attempts to correct or compensate for it with reinforced preventive measures (39). The present study also revealed that a relatively small number of dentists used a specialized form to conduct CRA (6.6%). While using such a form may be advantageous in the clinical setting, it still compared unfavorably to the 17% reported by Riley et al. for American and Scandinavian general practitioners who used a specialized form when assessing CR, and it was lower than the 31% of Japanese general practitioners who conducted CRA and used a form (10, 35). The finding that CRA form usage was higher among dentists trained in private institutions may indicate variability in CRA education and highlights the importance of integrating CRA more effectively into dental curricula.

Our findings emphasize the need for targeted educational interventions to improve the adoption of CRA among Guangzhou dentists. Promoting CRA can enhance the accuracy of caries risk identification and support more personalized preventive care. Moreover, incorporating digital technologies like electronic health records (HER) included within CRA modules (40), CRA software (16–19), or artificial intelligence (AI)-powered diagnostic tools (41), could smooth the CRA process for daily routine use in dental practitioners in Guangzhou. Use of these technologies can help in precise risk assessments, identify risk factors, and allow minimally invasive treatment planning for dental practitioners to better use of CRA into their daily practice, ultimately improving patient outcomes.

### 4.1 Limitations and strengths

The present study provides valuable insights into how dental practitioners in Guangzhou, China, knowledge, and practice CRA. However, it is important to consider several limitations of the study. Firstly, the online survey method employed may have introduced self-selection bias, as dental practitioners who were more inclined towards CRA may have been more likely to participate. Secondly, the data collected through self-reporting may have resulted in response bias, as some respondents may have provided inaccurate answers. Thirdly, another limitation could be the lack of detailed data on the specific dental specialist, while our study questionnaire determines both general dentists and specialists, but it did not determine more specific information about the department (e.g., restorative, pediatric, orthodontic). This gap could affect the findings of the present study, as the application of CRA might vary across different dental specialists. Therefore, future research could include variables to provide a detailed understanding of CRA covering different departments of dentistry. Finally, the study was geographically limited to dental practitioners in Guangzhou, and hence, the results may not be generalizable to other regions or countries with different dental practices and approaches to CRA. Despite these limitations, this study provides valuable information on the current adoption of CRA in Guangzhou, China. This is the first attempt to examine the knowledge and practices of dentists in Guangzhou with respect to CRA. The findings provide a comprehensive survey of the current situation regarding the use of CRA, which can help identify challenges to its adoption and promote the use of CRA techniques to reduce the prevalence of caries.

## 5 Conclusion

The present study indicates that dentists in Guangzhou mostly focus on various factors like oral hygiene, dietary habits, and active carious lesion, when evaluating CRA. However, there are limited use of standardized CRA forms and individual risk assessment in Guangzhou patients. Therefore, it requires the promotion of digital tools for CRA which include electronic health records with CRA modules or designated CRA assessment software that could improve CRA practices among dental practitioners in Guangzhou.

## Data Availability

The original contributions presented in the study are included in the article/Supplementary Material, further inquiries can be directed to the corresponding author.
